# When Medications Harm Muscles: A Case of a Teenager With Acute Rhabdomyolysis

**DOI:** 10.7759/cureus.101173

**Published:** 2026-01-09

**Authors:** Sari Aidek, Khalid Ibrahim, Mohamed Alaqqad, Ashraf Gumaa, Ahmed AbouHelwo

**Affiliations:** 1 Orthopaedics, Dubai Health, Dubai, ARE; 2 Radiology, Dubai Health, Dubai, ARE; 3 General Surgery, Hatta Hospital, Dubai Academic Health Corporation, Dubai, ARE; 4 Surgery, Hatta Hospital, Dubai Academic Health Corporation, Dubai, ARE; 5 Orthopaedic Surgery, Hatta Hospital, Dubai Academic Health Corporation, Dubai, ARE

**Keywords:** drug-induced myopathy, isotretinoin, medication-associated myopathy, muscle necrosis, rhabdomyolysis

## Abstract

Rhabdomyolysis is a rare but serious adverse effect of isotretinoin therapy, especially when combined with strenuous physical activity. We present a case of drug-induced rhabdomyolysis with secondary intramuscular abscesses in an adolescent. A 16-year-old male on standard-dose isotretinoin presented with a four-day history of right gluteal and thigh pain and fever, without a history of trauma, but with recent intense physical activity. Laboratory tests showed elevated inflammatory markers, creatine kinase, and myoglobinuria. MRI demonstrated extensive myositis with multiple intramuscular and intermuscular abscesses requiring hydration, antibiotics, drug discontinuation, and surgical drainage, leading to full recovery. This case emphasizes the need for early recognition of isotretinoin-associated rhabdomyolysis, particularly in physically active patients. Prompt imaging, cessation of the offending agent, and timely intervention can prevent renal injury and improve outcomes.

## Introduction

Rhabdomyolysis is a serious and potentially fatal condition marked by the rapid breakdown of skeletal muscle fibers, resulting in the release of intracellular contents, including electrolytes, myoglobin, and enzymes, into the bloodstream [1-3]. Its causes are typically divided into two broad categories. The first category includes physical triggers, such as direct trauma, vascular obstruction, prolonged immobilization, excessive physical exertion, electrical injuries, and hyperthermia. The second category comprises non-physical triggers, which include metabolic disorders, toxic exposures, certain medications (e.g., isotretinoin), infections, electrolyte disturbances, hormonal imbalances, and autoimmune myopathies such as polymyositis or dermatomyositis [4,5]. If undiagnosed or untreated, rhabdomyolysis can lead to severe complications, particularly hyperkalemia and acute kidney injury (AKI), both of which carry a high risk of mortality [6,7].

Among the medications implicated in rhabdomyolysis is isotretinoin, a first-generation retinoid derived from vitamin A. It is widely prescribed for various dermatologic conditions, including acne vulgaris and discoid lupus erythematosus. Isotretinoin is thought to induce apoptosis in certain cells capable of metabolizing it into all-trans retinoic acid, such as sebocytes, hepatocytes, intestinal epithelial cells, and myocytes [8].

Musculoskeletal complaints are among the most frequently reported adverse effects of isotretinoin [9]. These range from asymptomatic elevations in creatine kinase (CK) levels to more serious complications, such as localized or diffuse myositis, and, in severe cases, necrotizing myositis or rhabdomyolysis, which may require intensive care management [10].

## Case presentation

A 16-year-old previously healthy male presented to the emergency department with a four-day history of fever and right-sided gluteal and thigh pain. He denied any recent trauma; however, he was enrolled in a military school and reported regular physical activity. There was no personal or family history of neuromuscular disorders. One week before symptom onset, he had initiated oral isotretinoin at a standard therapeutic dose (0.5 mg/kg/day) for acne vulgaris.

On examination, the patient’s temperature was 37.2°C, and he appeared in pain, with tenderness over the lateral aspect of the proximal right thigh and gluteal region. The range of motion of the right hip was painful, and there was warmth and erythema over the lateral thigh and gluteal area. There were no neurological deficits, and distal circulation was intact.

Initial laboratory workup demonstrated marked inflammation and evidence of muscle injury (Table 1). Blood was taken and revealed no growth after five days.

**Table 1:** Initial laboratory investigations. WBC = white blood cell; ALT = alanine transaminase; AST = aspartate transaminase; ESR = erythrocyte sedimentation rate

Component	Result	Reference range and unit
WBC count	20.6	3.6–11 × 10³/µL
C-reactive protein	272.2	<5.0 mg/L
Creatine kinase (initial and peak)	564	0–270 U/L
Serum creatinine	0.7	0.70–1.20 mg/dL
ALT	136	0–41 U/L
AST	39	0–40 U/L
ESR	78	<20 mm/hour
Urinalysis	Positive for myoglobin	—
Procalcitonin	1.33	<0.05 ng/mL

Based on the clinical presentation of fever and focal muscle pain, along with elevated inflammatory markers and CK levels, an initial provisional diagnosis of septic arthritis, infective myositis versus early rhabdomyolysis was considered.

**Figure 1:**
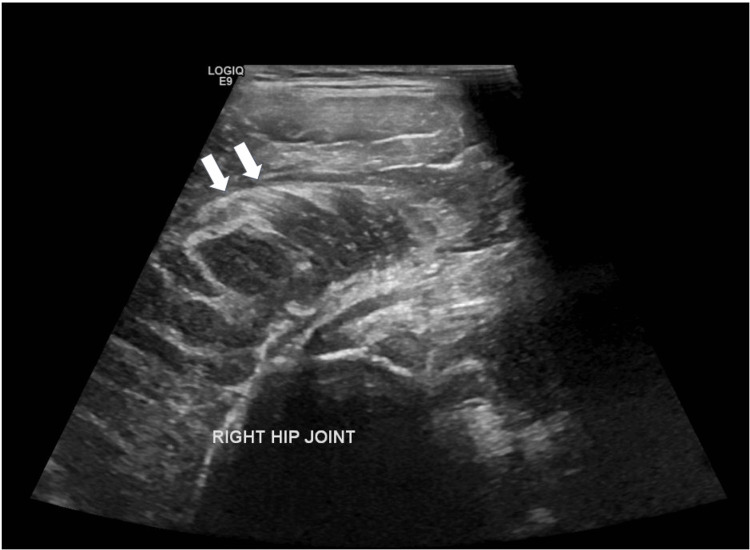
B-mode ultrasound of the right hip joint demonstrating a bulky gluteus minimus muscle (white arrows) with disorganized muscle fiber architecture.

An initial ultrasound of the right hip was performed to exclude septic arthritis. It demonstrated thickening of the gluteus minimus muscle with disrupted muscle fiber architecture (Figure 1).

**Figure 2:**
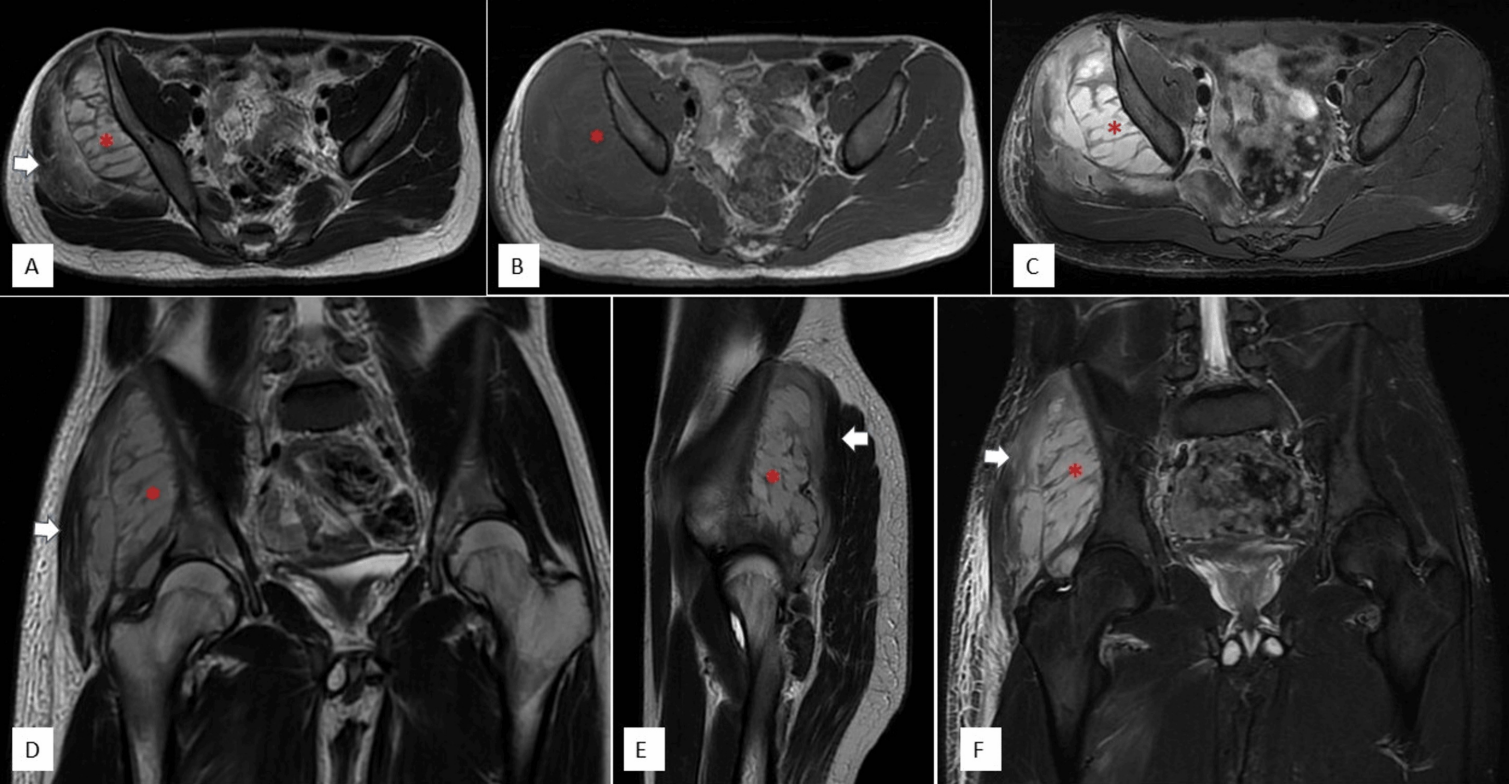
MRI of the pelvis demonstrating (A) axial T2, (B) axial T1, (C) axial T2 with fat suppression (water flex), (D) coronal T2, (E) sagittal T2, and (F) coronal short tau inversion recovery sequences. The right gluteus minimus muscle (red asterisk) appears bulky with extensive hyperintense signal on T2-weighted and short tau inversion recovery images, and iso- to hyperintense signal on T1-weighted images. Similar signal changes are noted in the adjacent gluteus medius (white arrow) and piriformis muscles, suggesting a myopathic or inflammatory process.

**Figure 3:**
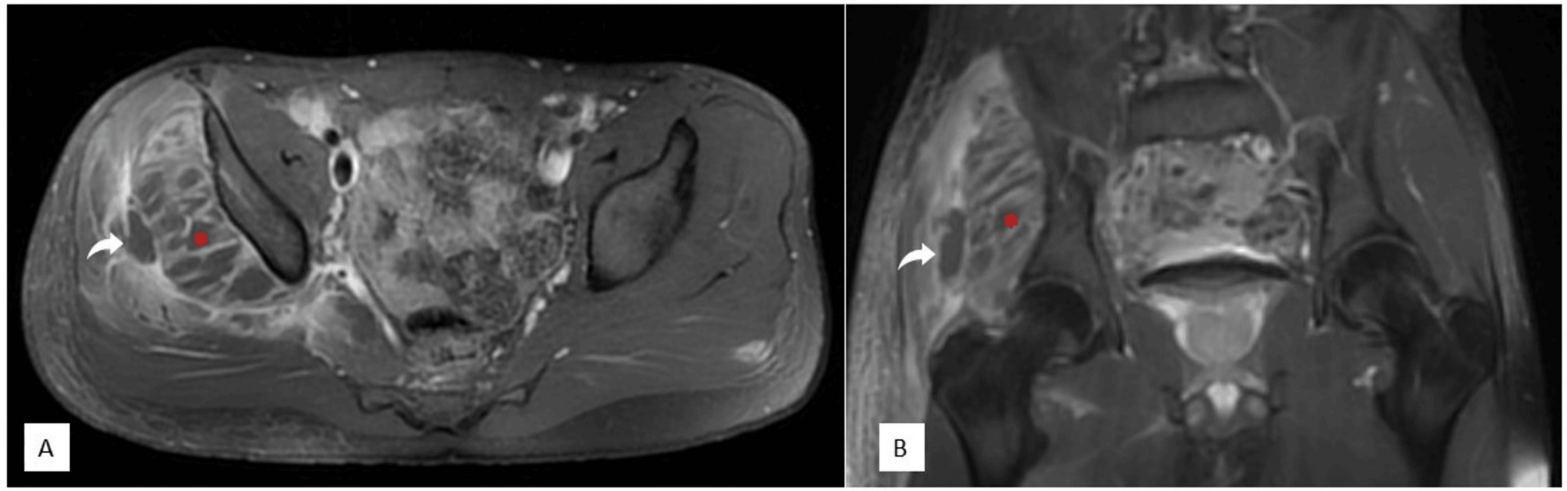
Post-contrast pelvis MRI showing (A) axial T1-weighted fat-suppressed and (B) coronal T1-weighted fat-suppressed images. The right gluteus minimus muscle (red asterisk) contains multiple small low-signal areas with peripheral enhancement, consistent with intramuscular fluid collections. A similar intermuscular collection is noted between the gluteus minimus and medius muscles (curved white arrow). Associated subcutaneous edema and enhancement are observed along the lateral aspect of the thigh. These findings are highly suggestive of an extensive inflammatory process with multiple intramuscular and intermuscular abscesses.

MRI of the pelvis revealed a bulky right gluteus minimus exhibiting extensive hyperintensity on both T2-weighted and short tau inversion recovery sequences, while presenting with iso- to hyperintensity on T1-weighted images. Similar involvement was noted in the gluteus medius and piriformis muscles. Additionally, multiple intramuscular low-signal foci demonstrated peripheral enhancement after contrast administration, consistent with the presence of abscesses. An intermuscular collection was observed between the gluteus minimus and medius, accompanied by associated subcutaneous edema and enhancement along the lateral thigh (Figures 2, 3). Collectively, these findings suggested a severe inflammatory myopathy with multiple intramuscular and intermuscular abscesses.

**Figure 4:**
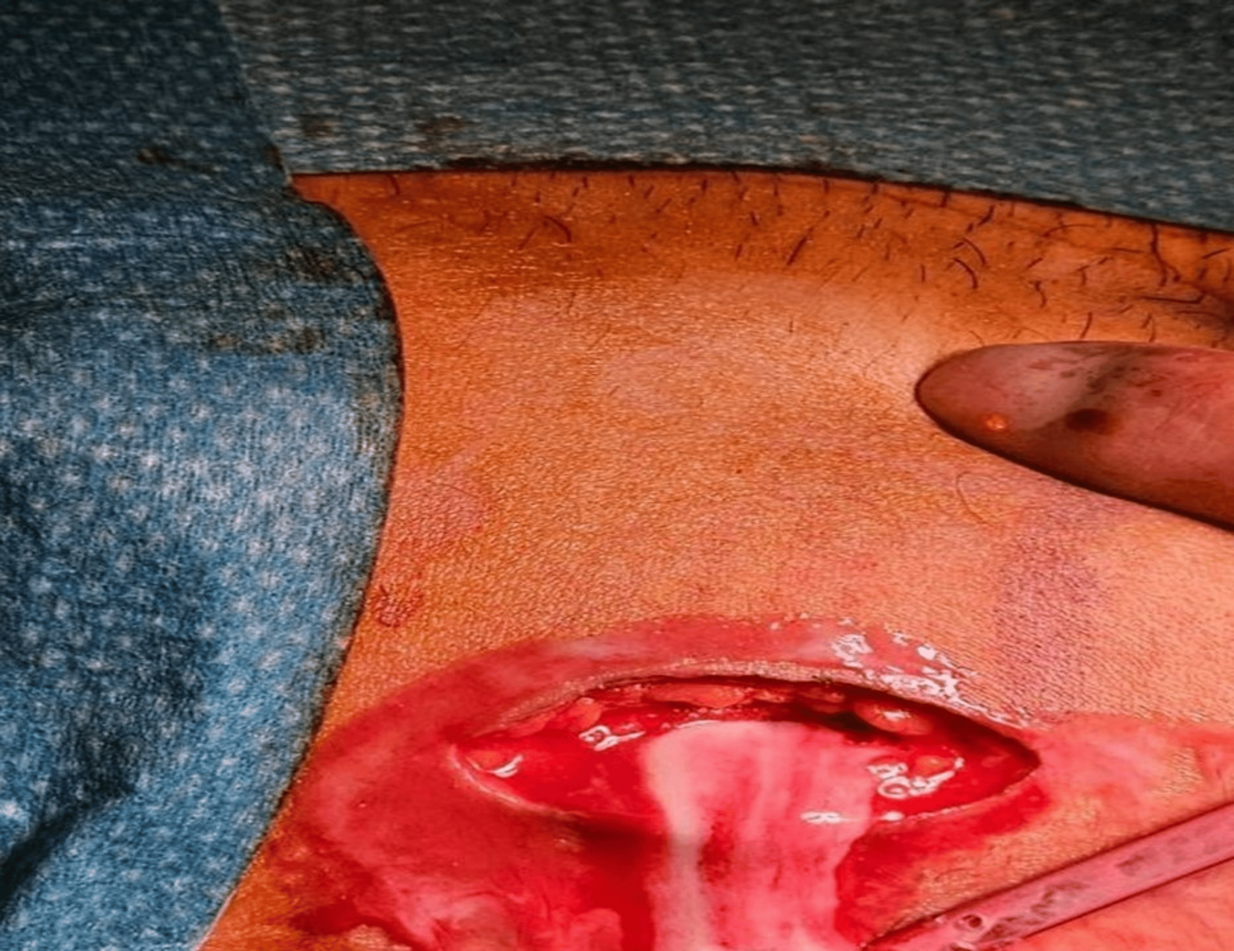
Intraoperative image demonstrating purulent fluid drainage through an incision at the anterolateral aspect of the right hip joint.

Isotretinoin was discontinued upon presentation. The patient received aggressive intravenous hydration for renal protection, electrolyte monitoring, and broad-spectrum intravenous antibiotics. Surgical drainage of the abscesses was performed. Open surgical drainage revealed a deep, multiloculated abscess involving the right gluteal musculature and extending to the lateral thigh, where 50 mL of pus was evacuated (Figure 4).

The drained pus was sent for culture and sensitivity, and the result revealed methicillin-resistant *Staphylococcus aureus* (moderate growth) sensitive to clindamycin, linezolid, rifampicin, trimethoprim + sulfamethoxazole, and vancomycin.

Muscle biopsy showed skeletal muscle fragments with moderate inflammatory infiltrate composed predominantly of lymphocytes, plasma cells, and occasional neutrophils, along with the presence of focal necrotic muscle fibers.

The triad of a temporal link to isotretinoin and exercise, biochemical evidence of muscle injury (CK elevation, myoglobinuria), and imaging/histologic confirmation of necrotizing myositis with abscess established isotretinoin-induced rhabdomyolysis with secondary infection. CK values, while modest (initial and peak 564 U/L), were diagnostic. Abscess formation contributed to the dominant presentation of fever and pain over profound CK elevation. MRI was critical in differentiating simple myositis from abscesses, guiding the transition to surgical intervention.

Over one week of hospitalization, the patient’s symptoms progressively improved. CK normalized (29 U/L on discharge), and renal function remained stable. He was discharged with counselling regarding the potential adverse effects of isotretinoin, including rhabdomyolysis. At the three-week follow-up, he demonstrated full recovery of hip range of motion with only mild residual reduction in gluteal muscle strength.

## Discussion

Rhabdomyolysis is a potentially life-threatening condition that occurs due to the rapid degradation of skeletal muscle fibers along with the release of intracellular contents, which can lead to electrolyte imbalances and AKI [11-13]. Typical clinical signs of rhabdomyolysis include muscle pain, weakness, and myoglobinuria (dark-colored urine), often accompanied by elevated CK levels, usually exceeding five times the upper limit of normal and frequently reaching over 1,000 U/L [14]. The majority of isotretinoin-associated cases have been reported in male patients [15]. Studies have shown that around 60% of rhabdomyolysis cases involve multiple contributing factors, and a synergistic effect may occur when isotretinoin use is combined with strenuous exercise, further elevating CK levels [11,16]. Several case reports have demonstrated that rhabdomyolysis is more likely to occur in patients receiving isotretinoin who engage in strenuous physical activity, including adolescents developing exertional rhabdomyolysis while on isotretinoin therapy [17,18]. This is consistent with our patient’s presentation, where initiation of isotretinoin combined with intense physical activity likely contributed to the development of rhabdomyolysis.

Although uncommon, isotretinoin may predispose patients to rhabdomyolysis through multiple mechanisms. The drug is believed to induce apoptosis in myocytes and other metabolically active cells [8], disrupt mitochondrial function, and elevate oxidative stress, thereby increasing the vulnerability of muscle fibers to injury during exertion [17]. Furthermore, isotretinoin can result in subclinical myopathy or asymptomatic CK elevations, which may synergize with physical stress to trigger clinically significant rhabdomyolysis [9,18].

AKI remains the most common systemic complication, occurring in 10% to 55% of cases, and its presence significantly worsens prognosis, particularly when multiorgan failure develops [13]. In rhabdomyolysis, AKI is mainly caused by myoglobin-induced renal tubular toxicity and impaired renal perfusion. Therefore, aggressive intravenous hydration is critical for maintaining renal perfusion and minimizing renal damage [19].

Effective management begins with identifying and eliminating the underlying cause. This may involve stopping a causative medication, correcting electrolyte imbalances, managing body temperature, or treating infections [20,21]. The prognosis of rhabdomyolysis is influenced by the underlying cause and coexisting conditions. Although large prospective studies are lacking, available case reports and small-scale studies suggest that, with timely and appropriate treatment, the outlook is generally favorable, and renal function often recovers fully. In our case, as the patient presented at an early stage, renal function was normal, and liver enzymes were only slightly elevated. Aggressive treatment was delivered to the patient, resulting in a good outcome.

## Conclusions

Rhabdomyolysis remains a major clinical challenge. Non-specific symptoms, multiple etiologies, and systemic complications obscure the diagnosis and complicate the treatment of this condition. This case highlights the importance of increased caution among clinicians prescribing medications with known muscle toxicity. Proper patient education regarding warning signs of rhabdomyolysis, particularly unexplained muscle pain, weakness, or dark-colored urine, can facilitate earlier detection and improve outcomes. We recommend that patients initiating isotretinoin therapy be advised to promptly report such symptoms, consider moderating intense physical activity at the start of therapy, and have a low threshold to check CK levels if symptoms arise. Timely recognition, cessation of the offending agent, and appropriate supportive interventions, including hydration and surgical management when indicated, can prevent renal injury and ensure favorable recovery.
